# Impact of Health Insurance Expansions on Nonelderly Adults With Hypertension

**DOI:** 10.5888/pcd12.150111

**Published:** 2015-07-02

**Authors:** Suhui Li, Brian K. Bruen, Paula M. Lantz, David Mendez

**Affiliations:** Author Affiliations: Brian K. Bruen, Paula M. Lantz, Department of Health Policy and Management, The George Washington University, Washington, DC; David Mendez, Department of Health Management and Policy, The University of Michigan, Ann Arbor, Michigan.

## Abstract

**Introduction:**

Hypertension is a risk factor for cardiovascular disease (CVD), the leading cause of death in the United States. The treatment and control of hypertension is inadequate, especially among patients without health insurance coverage. The Affordable Care Act offered an opportunity to improve hypertension management by increasing the number of people covered by insurance. This study predicts the long-term effects of improved hypertension treatment rates due to insurance expansions on the prevalence and mortality rates of CVD of nonelderly Americans with hypertension.

**Methods:**

We developed a state-transition model to simulate the lifetime health events of the population aged 25 to 64 years. We modeled the effects of insurance coverage expansions on the basis of published findings on the relationship between insurance coverage, use of antihypertensive medications, and CVD-related events and deaths.

**Results:**

The model projected that currently anticipated health insurance expansions would lead to a 5.1% increase in treatment rate among hypertensive patients. Such an increase in treatment rate is estimated to lead to 111,000 fewer new coronary heart disease events, 63,000 fewer stroke events, and 95,000 fewer CVD-related deaths by 2050. The estimated benefits were slightly greater for men than for women and were greater among nonwhite populations.

**Conclusion:**

Federal and state efforts to expand insurance coverage among nonelderly adults could yield significant health benefits in terms of CVD prevalence and mortality rates and narrow the racial/ethnic disparities in health outcomes for patients with hypertension.

## Introduction

In the United States, approximately 78 million people — or 1 in 3 adults — have hypertension, defined as systolic blood pressure of 140 mm Hg or higher or diastolic blood pressure of 90 mm Hg or higher (1). Hypertension is a risk factor for cardiovascular disease (CVD), contributing to 35% of myocardial infarctions (MIs) and strokes, and 49% of heart failures (2). It is estimated that a 5 mm Hg reduction of systolic blood pressure in the population would lead to a 9% to 14% reduction in CVD-related mortality rates (3). Thus, prevention of elevated blood pressure can avert many CVD-related deaths.

Despite the low cost of antihypertensive medications, there is inadequate management of blood pressure at the population level. National surveys conducted during 2011–2012 show that only 72% of people with hypertension were taking antihypertensive drugs, and 53% of hypertensive patients had their blood pressure under control (4). Lack of insurance coverage is a critical barrier to better treatment of hypertension. Compared with insured people with hypertension, uninsured people with hypertension are 4.4 times more likely to have an unmet need for medical care and prescription drugs (5) and have lower treatment and control rates (6).

Health insurance expansions under the Affordable Care Act (ACA) offered an opportunity to improve hypertension management by increasing the number of people receiving clinical preventive services (such as routine blood pressure checks) without cost sharing and by lowering patients’ out-of-pocket costs of antihypertensive medications. The Congressional Budget Office estimated that by 2024, Medicaid expansions and federal subsidies to buy insurance in the Health Insurance Marketplaces would help 25 million uninsured people get insurance coverage (7). However, little research has been done to understand the extent to which such expansion in coverage is likely to improve the health status of hypertensive patients in the long term. We aimed to project the long-term effects of health insurance expansions on hypertension treatment, CVD incidence rates, and disease-related mortality rates, using a state-transition (Markov process) model that simulates the lifetime health events among cohorts of the nonelderly hypertensive population.

## Methods

On the basis of empirical evidence that people with health insurance are more likely to receive antihypertensive medications and other medical interventions than those who are uninsured (8), we hypothesized that health insurance expansions would lead to fewer CVD events and related deaths among the hypertensive population. The goal of our model was to estimate changes in the incidence of stroke, coronary heart disease (CHD) — including MI and angina pectoris — and disease-caused mortality rates for a cohort of nonelderly adults, given the expected changes in health insurance rates and first-dollar coverage of preventive services among adults following implementation of the ACA. The simulation model ran separately for 8 discrete cohorts stratified by sex and age (in 10-year increments from 25 to 64). Previous research has used comparable approaches to project expected effects on CVD outcomes from changes in blood pressure or cholesterol levels as a result of prevention and treatment (9–11). However, given the well-established evidence that antihypertensive medications are highly effective in preventing CVD, we explicitly modeled health effects through improvement in the medication rate in the population.

Our simulation model estimated how changes in one input (ie, health insurance rate) lead to changes in other outputs (eg, incidence of CVD events) while isolating the effects of other confounding factors. Findings from this study can contribute to the understanding about the long-term impact of access to health insurance on the hypertensive population. The model also helps to assess changes in population health outcomes over time, complementing existing evidence from short-term retrospective data.

### Model design

Beginning with simulated year 2014 and at the start of each iteration (a calendar year), the model separates individuals in each cohort into 4 separate states: history of MI, history of angina, history of stroke, or “well” (ie, hypertension with no CVD history). In each simulated year, every individual may develop a CHD or stroke event, stay CVD-free, or die of a non-CVD cause; the probabilities of these events vary depending on whether an individual receives antihypertensive medications. Furthermore, each CVD event is associated with a certain probability of death, depending on the disease type and the patient’s age, insurance status, and disease history. Insurance status was randomly assigned at the beginning of each year, and the probability of insurance varied according to 3 policy scenarios.

**Figure 1:**
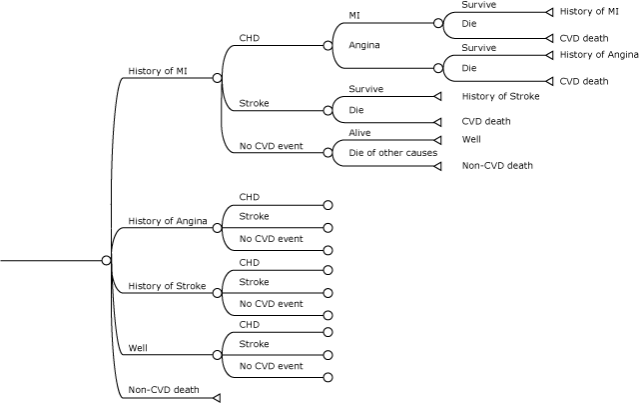
Simplified diagram of the Markov process. Abbreviation: CHD, coronary heart disease; CVD, cardiovascular disease; MI, myocardial infarction.

Figure 1 shows the state transitions for one piece of the model, where individuals start the year with a history of MI. At the end of each iteration, the model removes simulated deaths from each age–sex cohort, and the remaining lives carry on to the next simulated year. The lives that carry over also retain the history of cardiovascular outcomes acquired in the year that just completed, which changes the risk profile of the cohort (ie, the distribution of the 4 CVD risk groups) for the following year. This iterative process continues for the equivalent of 37 simulated years (2014–2050). Following the conclusion of the iterative model runs of each age–sex cohort, we computed the accumulated number of new CVD events and deaths until 2050. Finally, we computed the population-level CVD incidence and deaths by aggregating outcomes of all cohorts according to the age–sex distribution of hypertension prevalence (4).

### Policy scenarios and data input measures

We compared the population-level incidence of CVD and deaths by 2050 under 3 policy scenarios:

1. The baseline scenario simulated the absence of reforms included in the ACA, with percentages insured and uninsured remaining roughly the same as they were before enactment (12).
2. The first expansion scenario simulated an expansion based on current expectations (as of January 2014) for insurance expansions under the ACA and assumed that all undecided states will opt out of expanding Medicaid throughout 2050. Insurance coverage levels were based on research results reported by Nardin et al (13), which estimated that 13.9 million previously uninsured nonelderly adults would gain health insurance coverage under the ACA. These estimates were consistent with the observed decrease in the number of uninsured adults between 2013 and 2015 (14).
3. The second expansion scenario simulated an expansion that achieves 100% insurance for all age groups. This scenario goes beyond full implementation of the ACA, estimating the upper bounds on insurance-related effects that help to put the results from other scenarios into context.

The average annual incidence of CHD and stroke, by age and sex, was computed using β coefficients from Framingham CVD risk functions (15). The predictions of these functions have been validated with data from other ethnically diverse studies (16) and have been widely used in simulation models of CVD prevention strategies (10,17). Risk factors for CHD and stroke were derived from National Health and Nutrition Examination Survey 2011–2012 data, including sex, age, systolic blood pressure, smoking status, level of total serum cholesterol, level of high-density lipoprotein cholesterol, and the presence of diabetes. The distribution of CHD events (MI, stable angina, and unstable angina) was obtained from the Healthcare Cost and Utilization Project hospital inpatient data (18).

The likelihood of receiving antihypertensive medications by insurance status was based on estimates reported by Brooks et al (19) The age-specific effects of antihypertensive medications on CHD and stroke and 1-year mortality rates after CVD events were collected from published medical literature (12,13,20–30). (For complete information on input parameters, see the Appendix.)

We estimated the effects of health insurance expansions by race/ethnicity using the same process. Framingham CVD risk functions were used to estimate the probabilities of CVD events separately for each age-racial/ethnic group. For these scenarios, estimates of pre-ACA and post-ACA insurance rates by race/ethnicity were based on estimates reported by Clemans-Cope et al (31).

### Sensitivity analysis

We used Monte Carlo simulations to account for uncertainties about the disease transition probabilities at the individual level that could affect future outcomes. The probability of each CVD event was defined as a normally distributed random variable with means and standard deviations estimated from the Framingham CVD risk functions. The standard errors of disease prevalence and mortality rates were obtained from results of 1,000 simulations.

Past research found that longer periods without insurance are associated with access problems; thus, greater health benefits are likely to accrue for individuals with continuous coverage throughout their lifetimes (32). To test the effects of continuous coverage, we estimated an alternative model in which individuals in the same sex-age-insurance cohort remain either insured or uninsured for the duration of the simulation (Appendix). Additional analyses investigated the effects of including people with prehypertension (defined as systolic blood pressure of 120–139 mm Hg or diastolic blood pressure of 80–89 mm Hg). All analyses were performed using Treeage Pro 2013 (Treeage Software, Williamstown, Massachusetts).

## Results

Under the first expansion scenario, where all states currently undecided about Medicaid expansion opt out, the proportion of the current cohort of hypertensive patients being treated with antihypertensive medications is estimated to increase from 56.7% to 59.5% (Table 1). Younger adults would experience proportionally greater increase in treatment rates: rates for people aged 25 to 34 would increase by 9.4%, and rates for people aged 55 to 64 would increase by 3.4%. The changes in projected treatment rates were larger for men than for women, primarily because the current treatment rates for men are significantly below those for women. If all adults aged 25 to 64 obtained insurance coverage, the hypertension treatment rate would rise to 63.5%.

**Table 1:** Estimated Effects of Health Insurance Expansion on Hypertension Treatment Rates by 2016

Scenario/Sex	Nonelderly Adults With Hypertension, % Receiving Treatment (% Change Under Expansion)				
	All adults (N = 54,697,510)	Aged 25–34 (n = 3,911,740)	Aged 35–44 (n = 10,190,086)	Aged 45–54 (n = 17,326,094)	Aged 55–64 (n = 23,269,590)
**Baseline scenario: no expansion**					
US total	56.7 ( — )	51.5 ( — )	53.3 ( — )	57.0 ( — )	58.8 ( — )
Male	50.8 ( — )	45.9 ( — )	47.2 ( — )	51.6 ( — )	53.0 ( — )
Female	62.5 ( — )	58.8 ( — )	59.8 ( — )	62.8 ( — )	63.8 ( — )
**Scenario 1: currently undecided states opting out of Medicaid expansion**					
US total	59.5 (5.1)	56.3 (9.4)	56.9 (6.8)	60.2 (5.6)	60.7 (3.4)
Male	54.3 (6.8)	51.4 (12.1)	51.4 (8.9)	55.4 (7.2)	55.4 (4.5)
Female	64.8 (3.7)	62.7 (6.6)	62.7 (4.9)	65.4 (4.2)	65.4 (2.6)
**Scenario 2: all US population under insurance coverage**					
US total	63.5 (12.1)	62.9 (22.2)	63.3 (18.9)	63.4 (11.1)	63.8 (8.6)
Male	59.0 (16.1)	59.0 (28.6)	59.0 (25.0)	59.0 (14.3)	59.0 (11.4)
Female	68.0 (8.8)	68.0 (15.6)	68.0 (13.8)	68.0 (8.2)	68.0 (6.6)

The model predicted that if currently undecided states opt out of the Medicaid expansion and hypertension treatment rates remain constant, by 2050, the number of new cases of CHD would be reduced by 111,000 (0.55%) and the number of new cases of stroke by 63,000 (0.75%); the number of CVD-related deaths would decline by 95,000 (1.16%) (Table 2). If all currently uninsured people get insurance coverage, the incidence of CHD and stroke would decline by 1.48% and 1.3% respectively, and CVD-related mortality would decline by 2.73%. Both sexes were expected to benefit from the insurance expansions; the estimated benefits are greater for men than for women. Although the absolute numbers of new cases and deaths averted were estimated to be greater for the white population, the relative improvement in outcomes was expected to be greater for nonwhite Hispanics (Table 3). Relative to the baseline scenario, Hispanics were expected to have 2.07% fewer CHD cases and 1.06% fewer stroke cases under the first expansion scenario, compared with the 0.51% and 0.5% reductions for whites. The health effects of averted CVD events therefore translate into a greater mortality rate reduction for Hispanics than whites (3.84% vs 1.19%).

**Table 2:** Estimated Effects of Health Insurance Expansion on Nonelderly Adults With Hypertension, by Sex

Scenario/Sex	No. of CVD Events and CVD-Related Deaths Per 10,000 Population^a^		
	CHD	Stroke	CVD Death
**Baseline scenario: no expansion, no. (95% CI)**			
Total	2,022 (1,914–2,130)	837 (769–905)	816 (763–869)
Male	1,366 (1,303–1,429)	424 (391–457)	474 (446–502)
Female	648 (603–693)	421 (386–456)	342 (317–367)
**Scenario 1: currently undecided states opting out of Medicaid expansion**			
Total, no. (95% CI)	2,011 (1,906–2,116)	831 (763–899)	806 (753–859)
Difference (% change)	11.1 (−0.55)	6.3 (−0.75)	9.5 (−1.16)
Male, no. (95% CI)	1,358 (1,297–1,419)	420 (385–455)	468 (440–496)
Difference (% change)	8.3 (−0.61)	4.1 (−0.96)	5.8 (−1.23)
Female, no. (95% CI)	645 (596–694)	419 (379–459)	338 (303–373)
Difference (% change)	2.8 (−0.43)	2.3 (−0.54)	3.7 (−1.07)
**Scenario 2: all US population under insurance coverage**			
Total, no. (95% CI)	1,992 (1,884–2,100)	826 (761–891)	794 (744–844)
Difference (% change)	29.9 (−1.48)	10.9 (−1.30)	22.2 (−2.73)
Male, no. (95% CI)	1,344 (1,281–1,407)	418 (386–450)	460 (434–486)
Difference (% change)	21.9 (−1.6)	6.3 (−1.49)	14.0 (−2.96)
Female, no. (95% CI)	640 (590–690)	416 (377–455)	334 (299–369)
Difference (% change)	7.9 (−1.21)	4.8 (−1.14)	8.2 (−2.41)

Abbreviations: CHD, coronary heart disease; CI, confidence interval; CVD, cardiovascular disease.

a Values expressed as no. (95% CI), unless otherwise indicated. 95% CIs obtained from Monte Carlo simulations.

**Table 3:** Estimated Effects of Health Insurance Expansion on Nonelderly Adults with Hypertension, By Race/Ethnicity^a^^,^^b^

Scenario and Race/Ethnicity	CVD Events and CVD-Related Deaths Per 10,000 population		
	CHD	Stroke	CVD death
**Baseline scenario: no expansion, no. (95% CI)**			
White	1,310 (1,237–1,383)	534 (488–580)	528 (491–565)
Black	340 (321–359)	175 (162–188)	152 (143–161)
Nonwhite Hispanic	22.8 (21.6–24.0)	7.6 (6.9–8.3)	5.0 (4.5–5.5)
**Scenario 1: currently undecided states opting out of Medicaid expansion**			
White, no. (95% CI)	1,304 (1,231–1,377)	531 (485–577)	522 (487–557)
Difference (% change)	6.7 (−0.51)	2.7 (−0.5)	6.3 (−1.19)
Black, no. (95% CI)	336 (318–354)	173 (160–186)	148 (139–157)
Difference (% change)	3.5 (−1.04)	1.9 (−1.1)	3.2 (−2.14)
Nonwhite Hispanic, no. (95% CI)	22.4 (21.1–23.6)	7.5 (6.8–8.2)	4.8 (4.4–5.2)
Difference (% change)	0.5 (−2.07)	0.1 (−1.06)	0.2 (−3.84)
**Scenario 2: all US population under insurance coverage**			
White, no. (95% CI)	1,295 (1,223–1,367)	529 (484–574)	517 (482–552)
Difference (% change)	15.4 (−1.17)	4.6 (−0.87)	11.3 (−2.15)
Black, no. (95% CI)	332 (314–350)	172 (159–185)	146 (137–155)
Difference (% change)	7.2 (−2.12)	3.5 (−1.97)	6.1 (−4)
Nonwhite Hispanic, no. (95% CI)	21.6 (20.5–22.7)	7.4 (6.7–8.1)	4.5 (4.1–4.9)
Difference (% change)	1.3 (−5.63)	0.2 (−2.57)	0.5 (−9.79)

Abbreviations: CHD, coronary heart disease; CI, confidence interval; CVD, cardiovascular disease.

a Values expressed as no. (95% CI), unless otherwise indicated. 95% CIs obtained from Monte Carlo simulations.

b Pre- and post-ACA insurance rates for race/ethnicity groups were adjusted according to the age distribution of insurance status reported by The Kaiser Commission on Medicaid and the Uninsured (12).

**Figure 2:**
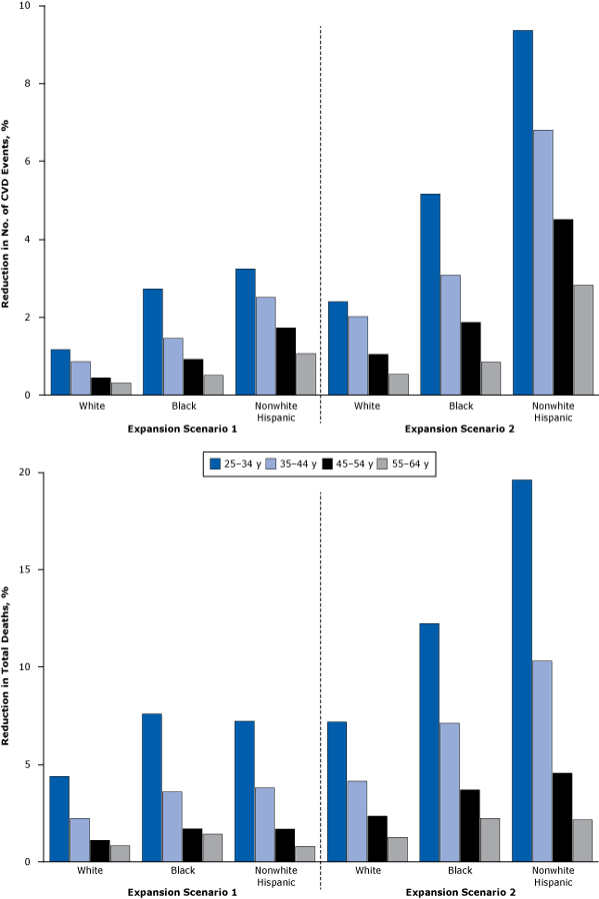
Estimated reduction in cardiovascular events and mortality rates under insurance expansions for white, black, and nonwhite Hispanic populations, by age group. These charts illustrate the racial/ethnic-specific effects of insurance coverage expansion by age group. Outcomes are measured by percentage reduction in cardiovascular events and mortality rates. Scenario 1 assumes currently undecided states opting out of Medicaid expansion, and scenario 2 assumes the entire US population is covered by insurance. **Table:** 

All age groups were expected to have reduced incidence of CVD events and lower mortality rates from CVD following the insurance expansions (Figure 2). The estimated reductions in mortality were largest for adults aged 25 to 34: by 1.2% to 3.2% if currently undecided states opt out of Medicaid expansions, and by 2.4% to 9.4% if all adults eventually have insurance coverage. Again, the estimated benefits are greater for nonwhite populations in each age group.

Scenario and Race/Ethnicity	Age, y			
	25–34 y	35–44 y	45–54 y	55–64 y
**Reduction in No. of CVD Events, %**				
**Expansion scenario 1**				
**White**	1.17	0.86	0.45	0.31
**Black**	2.73	1.46	0.92	0.51
**Nonwhite Hispanic**	3.24	2.51	1.73	1.07
**Expansion scenario 2**				
**White**	2.40	2.02	1.05	0.54
**Black**	5.16	3.08	1.87	0.85
**Nonwhite Hispanic**	9.36	6.80	4.51	2.82
**Reduction in Total Deaths, %**				
**Expansion scenario 1**				
**White**	4.39	2.23	1.11	0.83
**Black**	7.59	3.60	1.70	1.43
**Nonwhite Hispanic**	7.22	3.80	1.68	0.80
**Expansion scenario 2**				
**White**	7.19	4.15	2.35	1.27
**Black**	13.23	7.12	3.70	2.23
**Nonwhite Hispanic**	19.59	10.31	4.56	2.16

As expected, the model predicted greater health effects when individuals were assumed to maintain their insurance status over time (Appendix). In the first expansion scenario, improved medication rates would prevent 307,000 CHD cases (1.42%), 138,000 stroke cases (1.18%), and 217,000 CVD-related deaths (2.61%). In the second scenario, there would be 485,000 fewer CHD cases (2.24%), 266,000 fewer stroke cases (2.26%), and 513,000 fewer CVD-related deaths (6.15%).

When including all adults with prehypertension, the estimated relative effects of expansion remain similar to the baseline results (Appendix). Nevertheless, the predicted population-wide benefits in terms of the CVD cases averted and lives saved are greater, because an additional 53 million adults with prehypertension would benefit from early interventions due to coverage expansions. Specifically, the model projects that insurance expansions would reduce CHD cases by 253,000 to 535,000; stroke cases by 77,000 to 189,000; and deaths by 165,000 to 364,000.

## Discussion

One objective of the ACA is to improve disease prevention by expanding health insurance coverage and access to preventive care. Findings from this study indicate the potential public health benefits from such efforts. It is estimated that improved hypertension treatment rates due to insurance expansions would prevent 174,000 to 408,000 new CHD and stroke cases by 2050, a 0.61% to 1.43% decline from the baseline. Heidenreich et al (33) estimated that CHD and stroke cost $197.3 billion (2008 dollars) in 2015, including direct medical costs and indirect costs from illness and premature death. Applying these estimates to our results yields a cost savings of $1.2 to $2.8 billion per year. We also projected that increased hypertension treatment rates due to expansion would prevent 95,000 to 222,000 CVD-related deaths among the current cohort of nonelderly hypertensive patients, representing 2,568 to 6,000 lives saved annually.

These results build on findings from the lottery-based Medicaid expansion in Oregon, which indicated that previously uninsured low-income adults who were randomly selected into Medicaid reported increased use of medication for hypertension, hypercholesterolemia, and diabetes, and better self-reported physical and mental health, than low-income adults who remained uninsured (34). Nevertheless, significant improvement in clinical outcomes, such as blood pressure, cholesterol level, and glucose level, was not observed in the first 2 years following the expansion. As the authors acknowledged, the short study period may be a limiting factor, because the health benefits of having insurance may not be realized immediately. Thus, our results indicate that when considering whether to implement health insurance expansions, states should consider the population health benefits and cost-savings that would be realized in the long term.

Our estimate of a 1.2% to 2.7% reduction in population mortality is smaller than the estimate by Sommers et al of 6% (35). There are several explanations for this difference. Besides coverage for antihypertensive drugs, health insurance provides patients with timely outpatient care, chronic disease management, and laboratory services, all of which are likely to generate health benefits that are not captured in our model. Our estimates also exclude the health effects of insurance expansion on other conditions such as diabetes and mental illness. Given that the insurance coverage provision under the ACA is expected to cost $76 to $145 billion annually for the next decade (7), more research is needed to comprehensively evaluate the health and economic effects of this health system reform.

Our study has limitations. Our model predicted greater benefits for certain subpopulations. Men would receive greater benefits than women (eg, 1.23% decrease in mortality for men vs 1.07% for women in the first expansion scenario), because men have a higher lifetime risk of CVD (36). Young and middle-aged adults (25 to 54 y) would experience proportionally greater reductions in mortality rates, largely because early treatment of hypertension effectively prevents or delays the onset of CVD.

Our findings also suggest that the ACA’s insurance expansions would narrow the racial/ethnic disparities in health outcomes, a finding consistent with recent studies (35,37). In particular, nonwhite Hispanics would proportionally benefit the most from the coverage expansions because they have the lowest pre-ACA insurance rate among all racial/ethnic groups (31). Blacks would also receive proportionally larger benefits than whites, because they have the highest rates of hypertension as well as other CVD risk factors, such as diabetes and obesity (38).

There are several limitations of this analysis. As with almost all policy assessment tools, the size of our estimates depends on a range of assumptions about the population in future decades. As discussed previously, our analysis focuses on the impact of improved medication rates and does not fully consider other potential benefits from insurance expansions. Our main analysis also does not account for possible effects of health insurance expansions on preventing the onset of hypertension among healthy adults and adolescents — for example, potential reduction in hypertension prevalence because of lifestyle interventions and health education. As shown in the sensitivity analysis, insurance expansions also have positive effects on people with prehypertension. Conversely, factors that delay enrollment of uninsured individuals in Medicaid, insurance exchanges, or both, or that limit their ability to access care once insured, may lower the estimated effects.

Second, the estimates of baseline disease risks stratified by age, sex, and race/ethnicity are extrapolated from clinical-trial data collected in the 1980s, so any uncertainty about the effects of CVD risk factors on the current population would limit the accuracy of projections. Nevertheless, results from the fitted Framingham risk functions suggest that estimates of CVD risks are accurate, and Monte Carlo simulations show that small variations in baseline CVD risks have little impact on the estimated numbers of CVD events and deaths. However, the model did not account for year-to-year changes of CVD risks in the 10-year increments among cohorts or for future medical advances that may improve hypertension treatment efficacy. We were also unable to differentiate the burden of CVD disease in expansion and nonexpansion states.

Finally, another uncertainty concerns how health care reform affects the stability of individuals’ insurance coverage. Existing modeling approaches (eg, the Coronary Heart Disease Policy Model [11]) typically estimate the effect of a population-wide, constant reduction in health risk factors, such as sodium intake, on health outcomes. Unlike these models, we did not make explicit assumptions about individuals’ insurance status throughout their lifetimes. Instead, our estimation was driven by the mix of insured and uninsured among the current cohort of individuals aged 25 to 64, over future decades. Although our sensitivity analysis showed greater health effects when people have continuous insurance coverage, in practice, little is known about how often individuals drop insurance coverage and whether insurance reforms will shorten the average coverage gap. More research is needed to address these uncertainties.

Even with these limitations, this study demonstrates that improved hypertension treatment through the expansion of health insurance coverage would yield substantial health benefits for the 55 million nonelderly hypertensive adults in the United States. Future research should include additional analyses of the effects of comprehensive insurance benefit packages and improved blood pressure monitoring in home and ambulatory settings.

## Appendix.

Estimation of Model Input Parameters and Results of Sensitivity Analysis. This file is available for download as a Microsoft Word document.

## References

[1] Go AS , Mozaffarian D , Roger VL , Benjamin EJ , Berry JD , Blaha MJ , ; American Heart Association Statistics Committee and Stroke Statistics Subcommittee. Heart disease and stroke statistics—2014 update: a report from the American Heart Association. Circulation 2014;129(3):e28–292. 10.1161/01.cir.0000441139.02102.80 24352519 PMC5408159

[2] Padwal R , Straus SE , McAlister FA . Evidence based management of hypertension. Cardiovascular risk factors and their effects on the decision to treat hypertension: evidence based review. BMJ 2001;322(7292):977–80. 10.1136/bmj.322.7292.977 11312234 PMC1120139

[3] National High Blood Pressure Education Program Working Group. National High Blood Pressure Education Program Working Group report on primary prevention of hypertension. Arch Intern Med 1993;153(2):186–208. 10.1001/archinte.1993.00410020042003 8422207

[4] Centers for Disease Control and Prevention, National Center for Health Statistics. National Health and Nutrition Examination Survey, 2011–2012; 2014.

[5] Davidoff AJ . Uninsured Americans with chronic health conditions: key findings from the National Health Interview Survey; 2005. http://www.urban.org/uploadedpdf/411161_uninsured_americans.pdf. Accessed February 4, 2015.

[6] Centers for Disease Control and Prevention. Vital signs: prevalence, treatment, and control of hypertension—United States, 1999-2002 and 2005-2008. MMWR Morb Mortal Wkly Rep 2011;60(4):103–8. 21293325

[7] Congressional Budget Office. The budget and economic outlook: 2014 to 2024; 2014. http://www.cbo.gov/publication/45010. Accessed February 4, 2015.

[8] Duru OK , Vargas RB , Kermah D , Pan D , Norris KC . Health insurance status and hypertension monitoring and control in the United States. Am J Hypertens 2007;20(4):348–53. 10.1016/j.amjhyper.2006.11.007 17386339

[9] Grover SA , Abrahamowicz M , Joseph L , Brewer C , Coupal L , Suissa S . The benefits of treating hyperlipidemia to prevent coronary heart disease: estimating changes in life expectancy and morbidity. JAMA 1992;267(6):816–22. 10.1001/jama.1992.03480060062031 1732653

[10] Lovibond K , Jowett S , Barton P , Caulfield M , Heneghan C , Hobbs FD , Cost-effectiveness of options for the diagnosis of high blood pressure in primary care: a modelling study. Lancet 2011;378(9798):1219–30. 10.1016/S0140-6736(11)61184-7 21868086

[11] Bibbins-Domingo K , Chertow GM , Coxson PG , Moran A , Lightwood JM , Pletcher MJ , Projected effect of dietary salt reductions on future cardiovascular disease. N Engl J Med 2010;362(7):590–9. 10.1056/NEJMoa0907355 20089957 PMC3066566

[12] The Kaiser Commission on Medicaid and the Uninsured. The uninsured: a primer — key facts about health insurance on the eve of coverage expansion. 2013. http://kff.org/uninsured/report/the-uninsured-a-primer/. Accessed August 8, 2014.

[13] Nardin R , Zallman L , McCormick D , Woolhandler S , Himmelstein D . The uninsured after implementation of the Affordable Care Act: a demographic and geographic analysis. Health Affairs blog; 2013. http://healthaffairs.org/blog/2013/06/06/the-uninsured-after-implementation-of-the-affordable-care-act-a-demographic-and-geographic-analysis/. Accessed August 8, 2014.

[14] Office of the Assistant Secretary for Planning and Evaluation. Health insurance coverage and the Affordable Care Act; 2015. http://aspe.hhs.gov/health/reports/2015/uninsured_change/ib_uninsured_change.pdf. Accessed April 10, 2015.

[15] Anderson KM , Odell PM , Wilson PWF , Kannel WB . Cardiovascular disease risk profiles. Am Heart J 1991;121(1 Pt 2):293–8. 10.1016/0002-8703(91)90861-B 1985385

[16] D’Agostino RB Sr , Grundy S , Sullivan LM , Wilson P ; CHD Risk Prediction Group. Validation of the Framingham coronary heart disease prediction scores: results of a multiple ethnic groups investigation. JAMA 2001;286(2):180–7. 10.1001/jama.286.2.180 11448281

[17] Liew D , Lim SS , Bertram M , McNeil JJ , Vos T . A model for undertaking effectiveness and cost-effectiveness analyses of primary preventive strategies in cardiovascular disease. Eur J Cardiovasc Prev Rehabil 2006;13(4):515–22. 10.1097/01.hjr.0000224488.03221.97 16874139

[18] The Healthcare Cost and Utilization Project. National statistics on hospital stays (HCUPnet database). Agency for Healthcare Research and Quality; 2012. http://hcupnet.ahrq.gov/. Accessed August 8, 2014.

[19] Brooks EL , Preis SR , Hwang SJ , Murabito JM , Benjamin EJ , Kelly-Hayes M , Health insurance and cardiovascular disease risk factors. Am J Med 2010;123(8):741–7. 10.1016/j.amjmed.2010.02.013 20670729 PMC2913281

[20] Collins TC , Petersen NJ , Menke TJ , Souchek J , Foster W , Ashton CM . Short-term, intermediate-term, and long-term mortality in patients hospitalized for stroke. J Clin Epidemiol 2003;56(1):81–7. 10.1016/S0895-4356(02)00570-X 12589874

[21] Lee KL , Woodlief LH , Topol EJ , Weaver WD , Betriu A , Col J , ; GUSTO-I Investigators. Predictors of 30-day mortality in the era of reperfusion for acute myocardial infarction. Results from an international trial of 41,021 patients. Circulation 1995;91(6):1659–68. 7882472 10.1161/01.cir.91.6.1659

[22] Malmberg K , Yusuf S , Gerstein HC , Brown J , Zhao F , Hunt D , Impact of diabetes on long-term prognosis in patients with unstable angina and non-Q-wave myocardial infarction: results of the OASIS (Organization to Assess Strategies for Ischemic Syndromes) Registry. Circulation 2000;102(9):1014–9. 10.1161/01.CIR.102.9.1014 10961966

[23] National Heart, Lung, and Blood Institute. Morbidity and mortality: 2012 chart book on cardiovascular and lung diseases. Bethesda (MD): National Institutes of Health; 2012.

[24] Vaccarino V , Parsons L , Peterson ED , Rogers WJ , Kiefe CI , Canto J . Sex differences in mortality after acute myocardial infarction: changes from 1994 to 2006. Arch Intern Med 2009;169(19):1767–74. 10.1001/archinternmed.2009.332 19858434 PMC3113508

[25] Vernino S , Brown RD Jr , Sejvar JJ , Sicks JD , Petty GW , O’Fallon WM . Cause-specific mortality after first cerebral infarction: a population-based study. Stroke 2003;34(8):1828–32. 10.1161/01.STR.0000080534.98416.A0 12855836

[26] Whang W , Shimbo D , Kronish IM , Duvall WL , Julien H , Iyer P , Depressive symptoms and all-cause mortality in unstable angina pectoris (from the Coronary Psychosocial Evaluation Studies [COPES]). Am J Cardiol 2010;106(8):1104–7. 10.1016/j.amjcard.2010.06.015 20920647 PMC2950828

[27] Greenland P , Reicher-Reiss H , Goldbourt U , Behar S . In-hospital and 1-year mortality in 1,524 women after myocardial infarction. Comparison with 4,315 men. Circulation 1991;83(2):484–91. 10.1161/01.CIR.83.2.484 1991367

[28] O’Donoghue M , Boden WE , Braunwald E , Cannon CP , Clayton TC , de Winter RJ , Early invasive vs conservative treatment strategies in women and men with unstable angina and non-ST-segment elevation myocardial infarction: a meta-analysis. JAMA 2008;300(1):71–80. 10.1001/jama.300.1.71 18594042

[29] Law MR , Morris JK , Wald NJ . Use of blood pressure lowering drugs in the prevention of cardiovascular disease: meta-analysis of 147 randomised trials in the context of expectations from prospective epidemiological studies. BMJ 2009;338:b1665. 10.1136/bmj.b1665 19454737 PMC2684577

[30] Fowler-Brown A , Corbie-Smith G , Garrett J , Lurie N . Risk of cardiovascular events and death—does insurance matter? J Gen Intern Med 2007;22(4):502–7. 10.1007/s11606-007-0127-2 17372800 PMC1829431

[31] Clemans-Cope L , Kenney GM , Buettgens M , Carroll C , Blavin F . The Affordable Care Act’s coverage expansions will reduce differences in uninsurance rates by race and ethnicity. Health Aff (Millwood) 2012;31(5):920–30. 10.1377/hlthaff.2011.1086 22566430

[32] Sudano JJ Jr , Baker DW . Intermittent lack of health insurance coverage and use of preventive services. Am J Public Health 2003;93(1):130–7. 10.2105/AJPH.93.1.130 12511402 PMC1447707

[33] Heidenreich PA , Trogdon JG , Khavjou OA , Butler J , Dracup K , Ezekowitz MD , ; American Heart Association Advocacy Coordinating Committee; Stroke Council; Council on Cardiovascular Radiology and Intervention; Council on Clinical Cardiology; Council on Epidemiology and Prevention; Council on Arteriosclerosis; Thrombosis and Vascular Biology; Council on Cardiopulmonary; Critical Care; Perioperative and Resuscitation; Council on Cardiovascular Nursing; Council on the Kidney in Cardiovascular Disease; Council on Cardiovascular Surgery and Anesthesia, and Interdisciplinary Council on Quality of Care and Outcomes Research. Forecasting the future of cardiovascular disease in the United States: a policy statement from the American Heart Association. Circulation 2011;123(8):933–44. 10.1161/CIR.0b013e31820a55f5 21262990

[34] Baicker K , Taubman SL , Allen HL , Bernstein M , Gruber JH , Newhouse JP , ; Oregon Health Study Group. The Oregon experiment — effects of Medicaid on clinical outcomes. N Engl J Med 2013;368(18):1713–22. 10.1056/NEJMsa1212321 23635051 PMC3701298

[35] Sommers BD , Baicker K , Epstein AM . Mortality and access to care among adults after state Medicaid expansions. N Engl J Med 2012;367(11):1025–34. 10.1056/NEJMsa1202099 22830435

[36] Mackey RH , Belle SH , Courcoulas AP , Dakin GF , Deveney CW , Flum DR , ; Longitudinal Assessment of Bariatric Surgery Consortium Writing Group. Distribution of 10-year and lifetime predicted risk for cardiovascular disease prior to surgery in the longitudinal assessment of bariatric surgery-2 study. Am J Cardiol 2012;110(8):1130–7. 10.1016/j.amjcard.2012.05.054 22742719 PMC3462227

[37] Alegria M , Lin J , Chen CN , Duan N , Cook B , Meng XL . The impact of insurance coverage in diminishing racial and ethnic disparities in behavioral health services. Health Serv Res 2012;47(3 Pt 2):1322–44. 10.1111/j.1475-6773.2012.01403.x 22568675 PMC3418830

[38] Kurian AK , Cardarelli KM . Racial and ethnic differences in cardiovascular disease risk factors: a systematic review. Ethn Dis 2007;17(1):143–52. 17274224

