# Supplementary material for: Impact of Health Insurance Expansions on Nonelderly Adults With Hypertension
Source: Prev Chronic Dis. 2015 Jul 2;12:E105. doi: 10.5888/pcd12.150111 (PMC4512839; doi:10.5888/pcd12.150111)
Supplement: Supplementary file 1 [file 15_0111_Appendix.docx]

**Appendix**

**Estimation of hypertension prevalence**

We estimated the prevalence of hypertension by sex, race/ethnicity, and age using data from the National Health and Nutrition Examination Survey (NHANES), an annual survey of the health and nutritional status of the US population. The survey collects health information data through interviews and physical examinations. We identified a person with hypertension if the person self-reported use of medication for hypertension or had an average systolic blood pressure reading of more than 140 mm Hg or an average diastolic blood pressure reading of more than 90 mm Hg on examination.

**Estimation of CVD risk**

We applied findings from the Framingham Heart Study to the current population in order to estimate the baseline annual risks of cardiovascular disease (CVD). Designed to understand epidemiology of cardiovascular disease (CVD), the Framingham Heart Study enrolls participants free of CVD and cancer, measures their baseline characteristics, monitors major risk factors, and identifies CVD outcomes through long-term follow-ups. Several outcomes are measured on the basis of patient diagnostic codes, including coronary heart disease (CHD, including myocardial infarction, angina pectoris, and coronary insufficiency) and stroke (ischemic and hemorrhagic).

To predict the incidence of CHD and stroke, we used beta coefficients of Framingham CVD risk functions reported by Anderson et al (15). Risk factors were derived from the NHANES 2011–2012 data, including sex, age, systolic blood pressure (outcomes were not significantly changed when using diastolic blood pressure instead), smoking status, level of total serum cholesterol, level of high-density lipoprotein cholesterol, and the presence of diabetes.

The prediction of annual CHD and stroke probabilities took 3 steps. The first step was to estimate the individual-level 10-year probability of disease by inputting risk indicators into the risk equations:

Predicted 10-year probability of disease = $1-exp[-exp(\frac{\log\left( 10 \right)-\hat{u}}{\hat{\sigma}})]$.

For CHD risk,

$$\hat{u}=15.5305+28.4441\times female-1.4792\times\log\left( age \right)-14.4588\times\log\left( age \right)\times female+1.8515\times(log {\left( age \right))}^{2}\times female-0.9119\times\log\left( SBP \right)-0.2767\times smoking-0.7181\times\log\left( \frac{Total cholesterol}{HDL cholesterol} \right)-0.1759\times diabetes-0.1999\times diabetes\times female-0.1588\times ECG,$$

and $\hat{\sigma}=0.9145-0.2784\times\hat{u}$.

For stroke risk,

$$\hat{u}=26.5116+0.2619\times female-2.3741\times\log\left( age \right)-2.4643\times\log\left( SBP \right)-0.3914\times smoking-0.0229\times\log\left( \frac{Total cholesterol}{HDL cholesterol} \right)-0.3087\times diabetes-0.2627\times diabetes\times female-0.2355\times ECG,$$

and $\hat{\sigma}=-0.4312$.

Next, we computed annual disease risk for individuals: $Annual probability=1-\left( 1-{Prob}_{10yr} \right)^{\frac{1}{10}}$.

Finally, we restricted the sample to people with hypertension, and computed the weighted average of disease probabilities by sex, race/ethnicity, and age group. Because data concerning the prevalence of CVD in adults younger than 35 years were not readily available, the youngest cohorts (aged 25–34 y) were assumed to be free of CVD. For the remaining cohorts, we used the model estimates of 10-year accumulated incidence of CHD and stroke from the youngest cohorts as the baseline CVD prevalence rates.

**Sensitivity analysis**

For our main analysis, we did not make explicit assumptions about whether newly insured individuals keep their insurance coverage throughout their lifetime. To examine the extent to which continuous insurance coverage affects health outcomes, we estimated an alternative model, in which individuals in the same sex-age-insurance cohort remain either insured or uninsured for the duration of the simulation (there are 16 cohorts for 2 sexes, 4 age groups, and 2 insurance statuses). After the conclusion of iterative model runs of a cohort, we computed the accumulated probabilities of CVD events and death until 2050. We then estimated the number of CVD cases and deaths for each sex-age group by combining the insured and uninsured cohorts according to insurance rates. Finally, we computed the population-level number of CVD cases and deaths by aggregating outcomes of all cohorts according to the age-sex distribution of hypertension prevalence.

**Table 1. Model Input Measures**

| **Parameters** | **Data** | **Sources** |
| --- | --- | --- |
| **Prevalence of hypertension** | 8.0–62.1%^a^ | Estimated using NHANES 2011–2012 data |
| **Uninsurance rates** | | |
| Baseline scenario | 13.7–30.5%^a^ | The Kaiser Commission on Medicaid and the Uninsured (12) |
| Expansion scenario 1 | 8.5–17.6%^b^ | Nardin et al (13), Clemans–Cope et al, (31) |
| Expansion scenario 2 | 0% |  |
| **Treatment probabilities** | | |
| Probability of receiving medication if insured | 59% if aged below 65, 100% if aged 65 or above | Brooks et al (19) |
| Probability of receiving medication if uninsured | 16% if aged below 65, 100% if aged 65 or above | As above |
| **Incidence of cardiovascular disease** | | |
| Annual probability of CHD event | 0.04–2.7%^a^ | Calculated using Framingham CVD risk equations with risk factor profile (15) with risk factors obtained from NHANES 2011–2012 data |
| CHD event distribution | MI 89.4–96.1%; Unstable Angina 2.6–6.6%; Stable Angina 1.3–4.0% ^a^ | HCUP 2012 hospital utilization data (18) |
| Annual probability of stroke event | 0.02–1.0%^a^ | Calculated using Framingham CVD risk equations with risk factor profile (15) with risk factors obtained NHANES 2011–2012 data |
| RR of coronary heart disease events if treated | 0.61–0.75^b^ | Law et al (29) |
| RR of stroke events if treated | 0.5–0.75^b^ | As above |
| **CVD–caused mortality^c^** | | |
| One–year mortality after MI | 5.3–44.9%^a^ | Greenland et al (27) |
| RR of death after MI given MI history | 1.51 | Derived from coefficients of the CHD mortality risk model reported by Lee et al (21) |
| RR of death after MI given angina history | 1 | As above |
| RR of death after MI given stroke history | 1.41 | As above |
| One–year mortality after unstable angina^d^ | 2.4–8.0%^a^ | Calculated based on meta–analysis by O’Donoghue et al Age-mortality associations reported by Whang et al (26,28) |
| RR of death after angina given MI history | 1.31 | Malmberg et al (22) |
| RR of death after angina given angina history | 1 | As above |
| RR of death after angina given stroke history | 1.36 | As above |
| One–year mortality after stroke | 7.5–33.2%^a^ | Derived from Collins et al (20) |
| RR of death after stroke given MI history | 2.22 | Vernino et al (25) |
| RR of death after stroke given angina history | 0.66 | As above |
| RR of death after stroke given stroke history | 2.31 | As above |
| RR of death after CVD events given uninsurance | 1.26 | Fowler-Brown et al (30) |
| **Non-CVD mortality** | 0.09–7.43%^b^ | Calculated based on all–cause mortality rates reported by Nation Vital Statistics System 1999–2007 and the percent of CVD–related death reported by National Heart, Lung, and Blood Institute (23) |

Abbreviations: CVD, cardiovascular disease; CHD, coronary artery disease; MI, myocardial infarction; RR, relative risk.

^a^ Age-sex specific.

^b^ Age specific.

^c^ Mortality rates were calculated for age groups 25–34, 35–44, 45–54, 55–64, 65–74, 75–84, and 85 years or above.

^d^ Stable angina in absence of CHD or stroke history is assumed to have no direct association with 1–year mortality (15).

|  | **CVD Events and CVD-Related Deaths in 10,000 Population** | | |
| --- | --- | --- | --- |
|  | **CHD** | **Stroke** | **CVD Death** |
| **Baseline Scenario: No Expansion** | | | |
| US Total | 2,165±117 | 1,173±68 | 834±47 |
| Male | 1,418±62 | 607±26 | 492±21 |
| Female | 745±55 | 568±42 | 342±25 |
| **Scenario 1: Currently undecided states opting out of Medicaid expansion** | | | |
| US Total | 2,134±115 | 1,159±68 | 812±46 |
| Diff (% change) | -30.7 (-1.42) | -13.8 (-1.18) | -21.7 (-2.61) |
| Male | 1,393±60 | 598±26 | 478±21 |
| Diff (% change) | -25 (-1.77) | -8.9 (-1.47) | -13.5 (-2.75) |
| Female | 739±55 | 563±42 | 334±25 |
| Diff (% change) | -5.4 (-0.72) | -5.2 (-0.91) | -8.2 (-2.4) |
| **Scenario 2: All US population under insurance coverage** | | | |
| US Total | 2,117±114 | 1,146±67 | 783±44 |
| Diff (% change) | -48.5 (-2.24) | -26.6 (-2.26) | -51.3 (-6.15) |
| Male | 1,383±60 | 593±26 | 460±20 |
| Diff (% change) | -35.4 (-2.5) | -14.1 (-2.33) | -31.8 (-6.46) |
| Female | 732±54 | 556±41 | 323±24 |
| Diff (% change) | -12.9 (-1.74) | -12.6 (-2.21) | -19.6 (-5.72) |

**Table 2. Estimated Effects of Health Insurance Expansion, Assuming Continuous Insurance Coverage**

Abbreviations: CHD, coronary heart disease; CVD, cardiovascular disease; MI, myocardial infarction.

Note: Plus-minus values are 95% confidence intervals obtained from the Monte Carlo simulations.

**Table 3. Estimated Effects of Health Insurance Expansion, Including Pre-hypertensive Population**

|  | **CVD Events and CVD-Related Deaths in 10,000 Population** | | |
| --- | --- | --- | --- |
|  | **CHD** | **Stroke** | **CVD Death** |
| **Baseline Scenario: No Expansion** | | | |
| US Total | 4,289±213 | 1,026±102 | 1,563±95 |
| Male | 2,557±123 | 549±57 | 756±49 |
| Female | 1,722±45 | 487±35 | 807±25 |
| **Scenario 1: Currently undecided states opting out of Medicaid expansion** | | | |
| US Total | 4,263±213 | 1,019±104 | 1,547±95 |
| Diff (% change) | 25.3 (-0.59) | 7.7 (-0.75) | 16.5 (-1.05) |
| Male | 2,545±122 | 546±57 | 748±49 |
| Diff (% change) | 11.9 (-0.47) | 3.2 (-0.59) | 7.7 (-1.02) |
| Female | 1,709±44 | 483±34 | 798±25 |
| Diff (% change) | 13.3 (-0.77) | 4.5 (-0.93) | 8.8 (-1.09) |
| **Scenario 2: All US population under insurance coverage** | | | |
| US Total | 4,235±211 | 1,007±102 | 1,527±93 |
| Diff (% change) | 53.5 (-1.25) | 18.9 (-1.84) | 36.4 (-2.33) |
| Male | 2,525±121 | 540±55 | 736±48 |
| Diff (% change) | 31.1 (-1.22) | 9.2 (-1.68) | 20.1 (-2.65) |
| Female | 1,700±45 | 477±34 | 791±24 |
| Diff (% change) | 22.1 (-1.29) | 9.9 (-2.03) | 16.3 (-2.02) |

Abbreviations: CHD, coronary heart disease; CVD, cardiovascular disease; MI, myocardial infarction.

Note: Plus-minus values are 95% confidence intervals obtained from the Monte Carlo simulations.
